# The Design and Testing of a PEA Powered Ankle Prosthesis Driven by EHA

**DOI:** 10.3390/biomimetics7040234

**Published:** 2022-12-12

**Authors:** Qitao Huang, Bowen Li, Hongguang Xu

**Affiliations:** Department of Fluid Control and Automation Harbin Institute of Technology, Harbin 150001, China

**Keywords:** powered ankle prosthesis, electro-hydrostatic actuator (EHA), parallel elastic actuator (PEA), power output, gait

## Abstract

Several studies have shown that actuation concepts such as Serial elastic actuator (SEA) can reduce peak power and energy consumption in ankle prostheses. Proper selection and design of the actuation concepts is important to unlock the power source potential. In this work, the optimization design, mechanical design, control scheme, and bench experiments of a new powered ankle–foot prosthesis is proposed. The actuation concept of this prosthesis is parallel elastic actuator (PEA) composed of electro-hydrostatic actuator (EHA) as the power kernel and a unidirectional parallel spring as the auxiliary energy storage element. After the appropriate motor and transmission ratio was selected, a dynamic model of the PEA prosthesis was built to obtain the appropriate spring parameters driven by biological data. The design of the hydraulic and mechanical system and the controller were provided for the implementation of the designed system. Bench experiments were performed to verify the performance. The results showed that the designed prosthesis meets the biomechanical dynamics requirements. This result emphasizes the feasibility of the EHA as a power source and actuator and provides new ideas for the design of ankle–foot prostheses.

## 1. Introduction

Although the passive ankle prosthesis [1] can help the disabled to return a certain amount of energy to help them during walking, it does not have the ability of active output, which leads to greater energy consumption [2] and gait distortion in amputee patients during walking [3]. Compared with the knee prosthesis, designing a powered ankle prosthesis is much more challenging, largely due to the load characteristics of the ankle, which can require up to 1.6 ± 0.2 Nm/kg of peak torque and up to 2.5 W/kg of peak power for medium-speed level walking [4], while the knee joint in the walking process absorbs energy, so it hardly needs excessive power output of the knee prosthesis. Therefore, how to improve the power-volume/mass ratio has become a core topic in the field of dynamic lower limb research.

The concept of powered ankle prosthesis was first proposed by Herr [5], who demonstrated the effectiveness of powered prosthesis by measuring human metabolism [6]. Series elastic actuators (SEAs) [7] driven by motors has been the majority power kernel of powered ankle prosthesis. It has been proved that SEAs can amplify the power of the actuator by as much as 1.4 times under ideal load conditions [8]. In addition, SEAs can help enhance the force control ability of the actuator and reduce the shock load of the drive system, which are significant for gearboxes and screws [5]. Since then, a series of designs for ankle–foot prostheses have been proposed. SPARKy is ankle prosthesis with SEA powered by a 150 W DC motor designed by Sugar et al. in Arizona State University, USA. The prosthesis is enough to provide energy consumption and peak power output for an 80 kg subject walking at a speed of 1.8 m/s [9,10]. The later prototype SPARKy 3 introduces a pair of parallel mechanical Achilles tendons, which realizes the movement of ankle prosthesis in the coronal plane [11]. Grimmer et al. designed the Walk–Run ankle with SEA, powered by a 200 W DC motor [12]. The Walk–Run ankle was capable of providing 3 W/Kg in 1.6 m/s walking, 5.6 W/Kg in 2.6 m/s running, and the maximum torque was 2.1 Nm/kg. Herr added a unidirectional parallel spring based on SEA, so that SEA can choose lower spring stiffness to obtain better output characteristics while meeting the force bandwidth need of 3.5 Hz [5]. Their subsequent prototype of ankle and knee prosthesis TF-8 use SEA as their power kernel, which can provide a maximum torque of 160 Nm, a maximum velocity of 6 rad/s on its ankle joint, with an ankle segment mass of 2.6 Kg [13]. They concluded that the SEA can provide power compensation while adapting to more terrain.

Parallel elastic actuator (PEA) [14] is another actuation concept, which usually combines the motor with a unidirectional spring to share the force required by the motor. Compared with SEA, PEA can significantly reduce the torque requirement of the motor to the peak power requirement, without any change in speed, and contributes to the force bandwidth improvement of the entire drive system. Verstraten carried out an optimization analysis of the design of ankle–foot prosthesis, and the results show that the unidirectional parallel spring is the optimal result in terms of both power and energy consumption for their selected motor [15]. Frank designed a transfemoral prothesis with brushless DC motors of 200 W used in each joint. The parallel spring is used at the ankle joint to make up for the shortage of output torque. The hole prosthesis is 4.5 Kg, with a maximum ankle torque of 130 Nm and peak ankle power of 250 W, which can meet the needs of daily activities such as walking at medium speed and going up and down stairs for adults weighing about 80 kg [16]. However, PEA is not suitable for those prostheses using mechanical reducers such as gearbox and screw. The PEA, despite the introduction of springs, is a stiff driver. For the mechanical transmission devices (e.g., gear boxes, ball screws, etc.), the shock load during heal strike can cause damage. In addition, for those using mechanical transmission devices, installing force sensors on the end-effector is difficult, while SEA can transform force control to position control, which is more available for mechanical prostheses.

Electro-hydraulic actuator (EHA) is an alternative to the motor-mechanical reducer driver. EHA is a power-by-wire (PBW) servo system widely used in aerospace industry [17,18] which is appropriate for wearable robots due to its high output power to mass ratio, high controllability, and robustness. The University of Tokyo was the first to apply the EHA system to humanoid robots [19,20] and they demonstrated the advantages of the backdrivability of EHA over gear transmission in force control. Based on the Elan Foot passive hydraulic ankle–foot prosthesis produced by the company Blatchford, Dr. Yu from the University of Bath led the design of an electro-hydrostatic-powered ankle–foot prosthesis system, which can switch active and passive modes [21]. However, the hydraulic system of this prosthesis is energy-consuming and has a complex sealing structure. Wang designed an EHA-based prosthesis that is similar in function to AMP’s EEA [22]. It can charge the accumulator in advance and release it during the push-off phase. The simulation results show that the accumulator can provide 150 W peak power. Tessari designed a prototype of the EHA knee prosthesis [23] and he demonstrated the potential of the EHA system for high efficiency.

According to the literature review so far, EHA has been regarded as a pure hydraulic system, and its excellent performance as a transmission device has never been reflected in the design of prosthetic limbs. PEA also has greater potential in terms of power and energy consumption than SEA. This study attempts to combine EHA and PEA, and provide full play to both advantages via their combination. In our previous work [24], an EHA-driven ankle–foot prosthesis design was proposed where the SEA was pre-defined as the actuation concept of the prosthesis and the spring parameters were designed by the limitation of the force bandwidth. In this article, we combined EHA with a unidirectional spring and design the spring parameter with optimization to minimize the energy consumption in a single cycle.

The rest of the paper is organized as follows: Section 2 introduces the design of the ankle–foot prosthesis, including the design of hydraulic circuit and mechanical schematic, the part selection, and the parameter settings of the spring. Section 3 provides the prototype design, including the structure design and the control system. Section 4 is the experiment for validating the performance, and Section 5 is the discussion of the experimental results. Section 6 concludes this article.

## 2. Design Concept

### 2.1. Principal Design of the Mechanism and Hydraulic System

**Figure 1:**
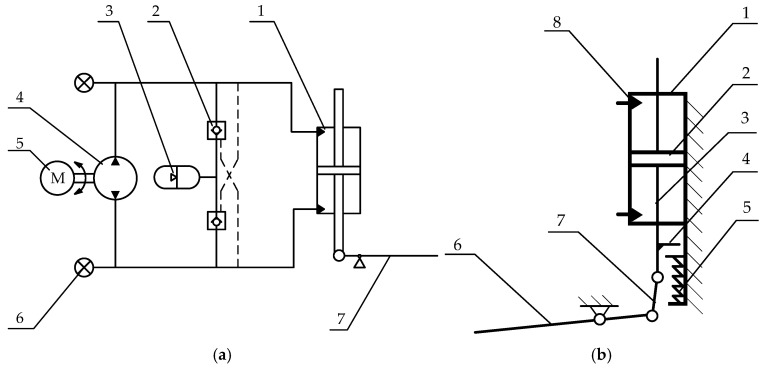
Schematic of the powered ankle prosthesis. (**a**) The hydraulic circuit diagram: 1: hydraulic cylinder; 2: check valve; 3: accumulator; 4: gear pump; 5: brushless DC motor; 6: pressure sensor; 7: sole; 8: link bar; 9: unidirectional spring. (**b**) The mechanical schematic: 1: hydraulic cylinder; 2: piston; 3: piston rod; 4: shaft shoulder on piston rod; 5: unidirectional spring; 6: sole; 7: link bar; 8: piping to the oil supply unit.

PEA has a greater ability to reduce output demand than SEA, and electro-hydraulic direct drive has the inherent advantage of attenuating impact loads. Thus, we chose to use PEA with EHA as the overall driving core. The schematic of the powered ankle prosthesis is shown in Figure 1. In the hydraulic circuit, a brushless DC motor and a bi-directional gear pump were used to deliver hydraulic oil directly to either side of the ankle cylinder, while no damping valve was added to avoid unnecessary pressure loss. The accumulator charges energy to the low-pressure side through the check valve of the circuit. The hydraulic circuit was pre-charged with pressure to prevent cavitation. The mechanical schematic is shown in Figure 1b. A four-bar mechanism was designed to achieve single degree of freedom rotation in the sagittal plane of the ankle joint. A unidirectional parallel spring was mounted under the hydraulic cylinder to share the load force when the cylinder’s piston is moving beyond the equilibrium position.

For walking between 0.5 and 2.6 m/s, the abled body angular velocity range is −250–320°/s, the maximum joint moment is 1.6 Nm/Kg, and the maximum angular range of motion of the is 40° [12,25]. The swing phase duration is changed with the step speed, as the speed increases the swing phase time percentage increases according to the research and product swing phase time [1,4], the maximum swing phase time set in this paper is 0.4 s. In summary, all the parameter index settings are shown in the Table 1 which were designed to meet the level ground walking requirements of an adult male of average height walking at a normal speed (1.25 m/s).

In order to select the best scheme to reduce the size and mass of the prosthesis with meeting the basic performance, the selection of each component and parameters on the overall design must be strictly considered. Design needs to start with the selection of the motor and then other parts of the prosthesis can be designed around the motor performance. The motor is not an ideal torque source, so the dynamic simulation is also necessary during the designing period.

### 2.2. Motor Selection

The motor should allow the inertia to be distributed along the leg rather than perpendicular to it, so torque motors were excluded. Thus, high speed motors with smaller diameter less than 40 cm were selected while the mass was less than 500 g.

With the same volume and mass, the most efficient motor is selected. The additional power consumption of the motor mainly comes from heating, which is generated by the current due to the load and bearing friction. The motor with the lowest heating power was selected. In the case of sufficient drive capacity, the performance of the motor is mainly limited by the heat, which is determined by the maximum continuous current and the motor driver limits the RMS of the current to protect the motor from overheating. Thus, we calculate the heating power of the motor by the continuous maximum current value $I_{cont}$ and motor terminal resistance R as (1).

(1) $$P_{H}=I_{cont}{}^{2}R$$

In addition, the acceleration performance of the motor also needs to be considered. The PEA does not help motor reduce speed throughout the cycle, so choosing a motor with low inertia is significant. According to the above criteria, we select the appropriate motor in Maxon Motor. The EC-I 40 brushless DC motor (100 W) was selected and parameters are shown in the Table 2.

### 2.3. Transmission Ratio

The speed requirement of the ankle prosthesis for walking task is 4 rad/s (38.2 r/min), the rated no-load speed of the motor is 5000 r/min, and the maximum allowable speed of the machine is 8000 r/min. As the motor is the highest efficiency in the working state of high speed and low torque, we attempted to choose a higher transmission ratio to make the motor work in the high efficiency range. Under the condition of satisfying the speed performance, we preliminarily selected 100 as the transmission ratio of the system (from the motor shaft to the ankle joint rotation pair) with consideration of volume efficiency of the hydraulic pump.

### 2.4. Spring

#### 2.4.1. Driver System Modeling

As mentioned above, the purpose of the spring is to enable the entire drive system to meet the power requirements within the limits of the motor. Therefore, we need to model the motor dynamic system and analyze it under the specified load and the spring parameters were selected based on the result.

The motor model was established by voltage Equation (2) and torque Equation (3). Where $J_{p}$ is the inertia of the pump, ${\dot{\theta }}_{m}$ is the motor speed, $T_{m}$ is the load torque on the motor, U is the motor voltage, and I is the motor current.

(2) $$k_{t}I=((J_{m}+J_{p}){\ddot{\theta }}_{m}+T_{m}+v_{m}{\dot{\theta }}_{m})$$

(3) $$U=L\dot{I}+RI+k_{b}{\dot{\theta }}_{m}$$

The torque at both ends of the transmission device can be expressed by (4). Transmission efficiency needs to be considered. The efficiency of working in quadrant 1, 3 should be distinguished from that in quadrant 2, 4. This model does not consider nonlinear effects, and the same efficiency was selected, which is represented by (5). Where the $T_{l}$ is the load joint torque, *C* is the efficiency coefficient, and N is the transmission ratio.

(4) $$T_{m}=\frac{C}{N}T_{l}$$

(5) $$C=\{\begin{array}{c} 1/\eta \;\;\;\;(P_{m}>0)\\ \eta \;\;\;\;\;\;\;\;(P_{m}<0) \end{array}$$

A unidirectional parallel spring can be represented by (6), where $k_{p}$ is the spring stiffness, $T_{s}$ is the torque equivalent torque on the parallel spring, and $\theta_{eq}$ is the equilibrium point of the spring. The spring is only engaged when the ankle angle is greater than $\theta_{eq}$.

(6) $$T_{s}=\{\begin{array}{cc} k_{p}(\theta -\theta_{eq}) & \left(\theta \geq \theta_{ini}\right)\\ 0\;\;\;\;\;\;\;\;\; & (\theta <0) \end{array}$$

The torque provided by the motor is equal to the load torque minus the part shared by the spring as (7), where T is the ankle torque.

(7) $$T=T_{l}-T_{s}$$

#### 2.4.2. Limits and the Indicator of the Optimization

The constraints of the motor mainly come from overspeed and overcurrent.

Overspeed can lead to reduced bearing lifetime of the motor or cause overvoltage of the motor drive due to back electromotive force. If the gear ratio is properly designed, the former almost never happens. Restrictions usually come from the diver voltage limit, where $U_{\max}$ is the maximum output of the motor driver.

(8) $$U\leq U_{\max}$$

Overcurrent can cause long-term and short-term overheating of the motor. Drivers limit the RMS of the current to limit long-term overheating, short-term overheating is usually limited by the output capacity of the driver itself. The motor current I should not exceed the driver maximum current $I_{\max}$ at any time, and the effective value should not exceed continuous operating current $I_{cont}$ as shown in Equations (9) and (10). The set of parameters that leads to the lowest power consumption as described with (11) is used for the final design. The finding method is global research. Driver limit parameters and transmission parameters related to the simulation are shown in Table 3.

(9) $$\left|I\right|\leq I_{\max}$$

(10) $$\sqrt{\frac{1}{T}\int_{0}^{T}I^{2}dt}\leq I_{cont}$$

(11) $$\int_{0}^{T}\left|U\cdot I\right|dt$$

According to the model and constraints proposed above, we excluded the spring parameters that did not meet the requirements with the global research method, and selected the combination of spring parameters that minimized the energy consumption of the motor as the elastic energy storage device.

#### 2.4.3. The Optimization Results

**Figure 2:**
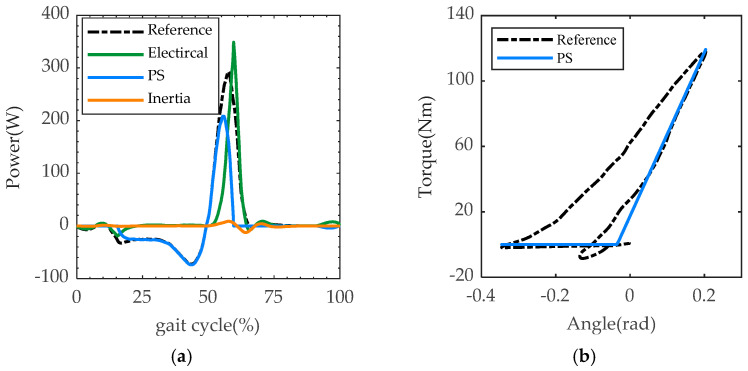
Simulation result of the optimization. (**a**) Power results of the simulation, where the reference power (black), motor power (green) and parallel spring power (blue) are plotted along the gait cycle. (**b**) Reference data of the able-body (black) from [4] and the optimal result of the parallel spring stiffness (blue).

As the optimization results show in Figure 2 and Table 4, the stiffness curve of the parallel spring is close to one side of the quasi-stiffness curve of the sample data. This makes sense because the optimization goal was to minimize the energy consumption, and the motor only needs to compensate the power represented by the sample containment area. At the same time, the parallel spring with the optimized result hardly affects the reset of the swing phase, so the position control of the swing phase is not affected. Compared with the driving system without spring, the optimized results can reduce the peak electrical power to 348.6 W (from 524.7 W) and the electrical energy consumption to 18.3 J (from 64.2 J) in a single gait cycle.

Direct drive (DD) is the case without any elastic element. In the case of optimization with electrical energy consumption, $k_{p}$ was 500 and $\theta_{eq}$ was −2°. In each gait cycle, the peak power of the motor driver $P_{diver}$ was 348.6 W, the peak power provided by the parallel spring $P_{PS}$ was 208.3 W, and the motor driver consumes electrical energy $E_{diver}$ of 18.3 J. Compared with the case without the parallel spring, this energy consumption $E_{DD}$ and electrical peak power $P_{DD}$ were 64.2 J and 524.7 W, respectively.

## 3. Specific Implementation

### 3.1. The Overall Design

**Figure 3:**
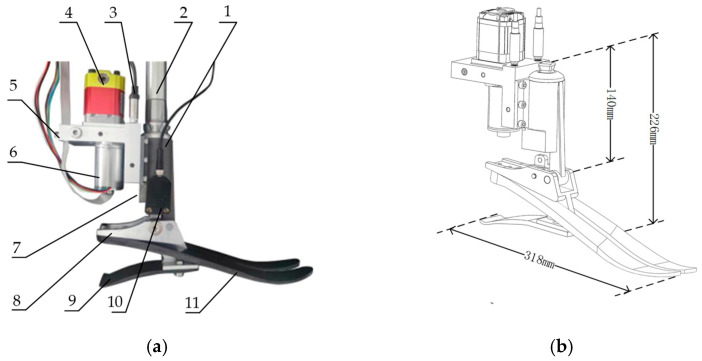
The ankle prosthesis. (**a**) The overall design of the prosthesis: 1: hydraulic cylinder; 2: shank link; 3: pressure sensors; 4: gear pump; 5: accumulator connector; 6: brushless DC motor; 7: spring slot; 8: connector; 9: heel spring; 10: displacement transducer; 11: sole. (**b**) The structure and the key dimension of the prosthesis.

The whole design is shown in Figure 3. The height of the prosthesis is 226 mm (from the bottom of the foot to the pyramid adapter), the height from the joint to the adapter is 140 mm, and the length of the foot is 318 mm. The range of motion of the prosthesis is 40° (with 20° on both sides of the foot in the flat position). The hydraulic cylinder and its internal components, the valve block, and the foot joint are fabricated of aluminum, and the foot as well as the heel are fabricated of carbon fiber. The overall mass of the prosthesis is 2.6 kg. The brushless DC motor and bidirectional gear pump were used to deliver hydraulic oil directly to both sides of the ankle cylinder through very short and thick pipes integrated inside the manifold block. The hydraulic cylinder was integrated in the shank to minimize the volume of the prosthesis. A four-bar linkage transforms the linear displacement into angular displacement. The unidirectional parallel spring was mounted in the slot of the cylinder. The designed parameters and parts considered in the criterion above are shown in Table 5.

### 3.2. Control System

The control program was on a National Instruments (NIs) CompactRIO system via add-on modules where two independent ADC modules (NI 9381, NI 9207) were used to acquire data from all the sensors and the motor-pump unit was powered by a 48 V lithium-ion battery through an amplifier (Escon 50/5 Servo Controller) which can record the motor speed and the drive current in the meantime. An angular sensor was used to measure the ankle rotation angle about the joint while two pressure transducers were mounted at the outlets of pump to measure the hydraulic pressure on both sides of the hydraulic cylinder. The whole electronic system is temporarily powered by wall socket power supply and will be replaced by a lithium battery in future research.

**Figure 4:**
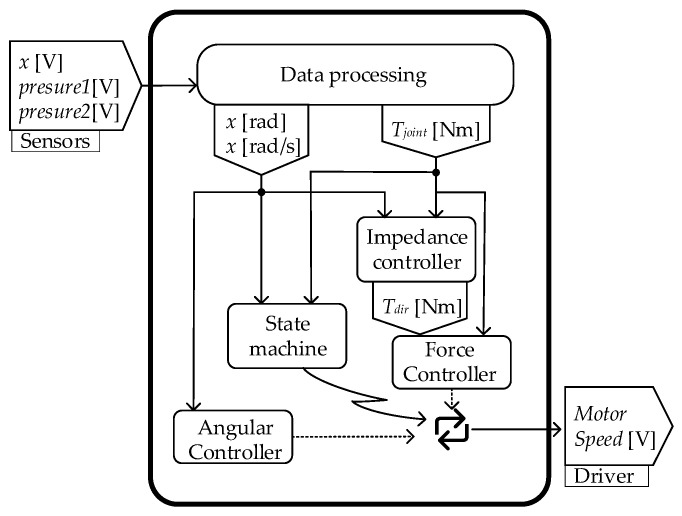
The controller flow chat. The control system takes the voltage signal from the sensors as inputs and outputs the voltage signal for the speed command to the motor driver.

**Figure 5:**
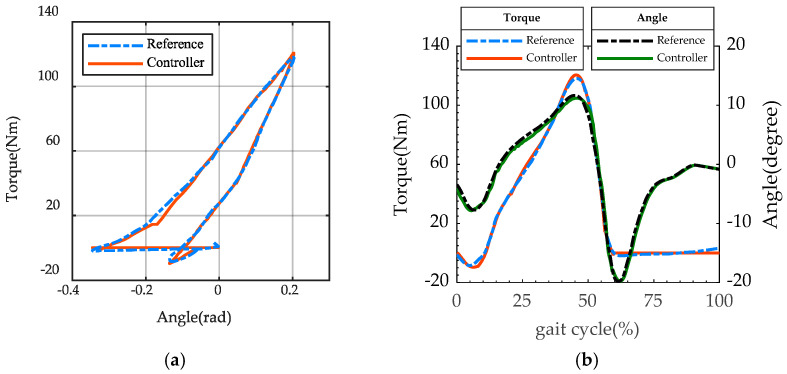
Simulation result of the optimization. (**a**) Simulative and reference quasi-stiffness. (**b**) Simulative ankle joint torque and angle with reference data.

The controller flowchart is shown as Figure 4. Firstly, the raw data of position sensors and pressure sensors are filtered and converted to joint angle and joint torque. Secondly, the state machine switches the support phase and swing phase according to the processed data. Then, during the stance phase, the impedance controller outputs a torque command based on the processed data, and the PD force controller is used to follow the command. The reset command of the sole is issued by the position controller and followed during the swing phase. Finally, the output of the force controller and position controller is converted into a voltage signal for the motor driver. The specific control algorithm and the modeling of the whole EHA system were described in detail in [24,26]. The controller accurately reproduces the biomechanical quasi-static stiffness profile as show in Figure 5a, which is important for ankle–foot prostheses. At the same time, the forces and angles were restored to a larger extent throughout the gait cycle compared with the biomechanical data, as shown in Figure 5b.

## 4. Experiments

### 4.1. Fast Response Test

Before proceeding to clinical trials, it is necessary to verify the performance of the ankle–foot prosthesis. We tested the stance phase and the swing phase separately.

**Figure 6:**
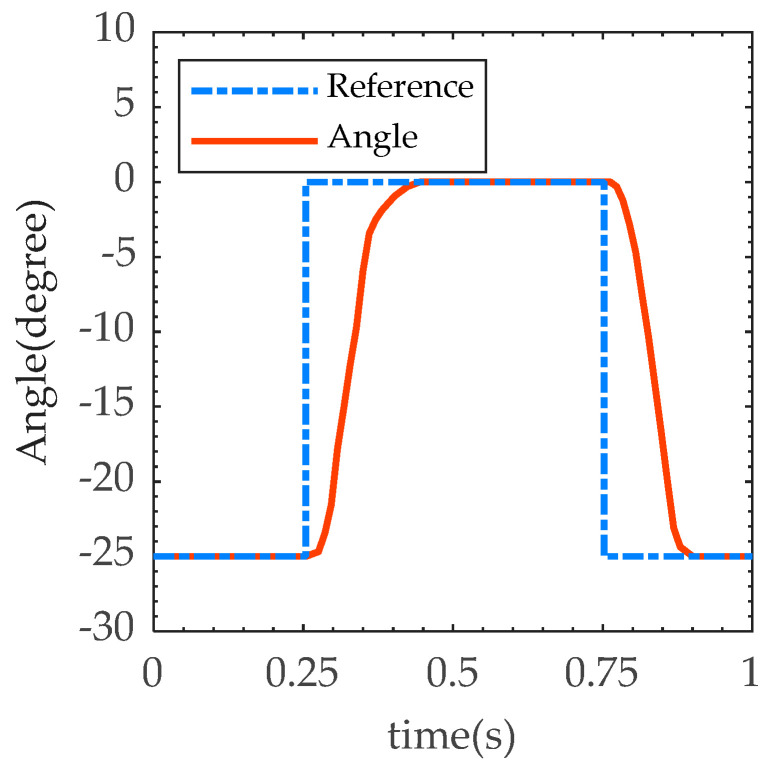
Result of fast response test.

There is no external load during the swing phase, which is only the position control of the EHA system. The sole of the foot should be repositioned quickly enough to prevent falls and be ready for the next heel strike. The maximum motor speed was set to 5000 RPM and the maximum current limit was 15 A. For level walking, we set 25° as the range of motion for dorsiflexion at the swing phase. Step command with amplitude of 25° was set to adjust PD parameters for fast response without overshoot. The result is shown in Figure 6. The step response time of the prosthesis was 0.2 s (rise time 0.15 s, fall time 0.12 s), which meets the rapidity requirement of the swing phase period as provided in Table 1.

### 4.2. Loading Test

**Figure 7:**
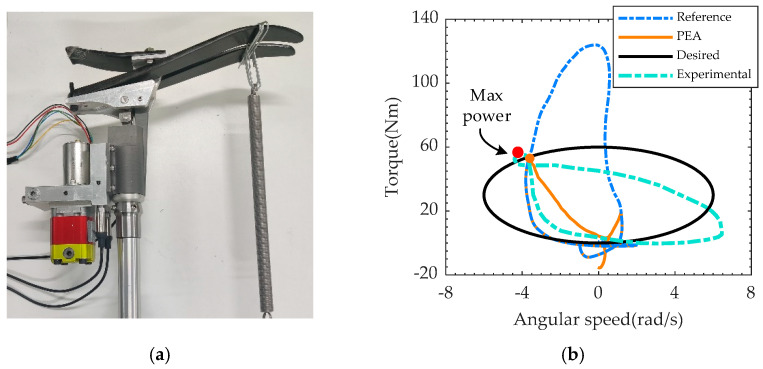
The loading test device and the load trajectory: (**a**) the experimental device of the loading test; (**b**) load trajectory expressed by angular speed and torque.

The load trajectory of stance phase can be represented by the torque–velocity curve shown in Figure 7. The blue line is the load trajectory of an able body while the red curve is the load curve of PEA and the black line is the load trajectory under the execution of sinusoidal position commands with spring load. In order to verify the capability of EHA system designed in Chapter 3, an elastic load is designed as (12) and the ankle should move as (13)

(12) $$T_{l}=-k_{l}(\theta +\theta_{o})$$

(13) $$\theta =\theta_{0}\sin\omega t$$

where $k_{l}$ is the stiffness of the spring load, $\theta_{o}$ is the equilibrium point.

These parameters were set as Table 6 to cover the motor load trajectory of the PEA, as depicted as orange line in Figure 7. The blue curve in Figure 7b is the torque–velocity curve of the ankle joint of a 75 kg able-body male with a maximum speed of 4 rad/s and a maximum torque of 125 Nm. The orange curve is the load curve of the EHA of the PEA prosthesis, which is the power that needs to be provided by the driver in addition to the power provided by the parallel spring. According to the load trajectory, if the EHA system can provide a load greater than the orange load curve, it proves that the prosthesis can meet the human body requirements corresponding to the blue curve. If the EHA system can meet the elastic load requirements within the limits described in Section 2.4.2, it also meets the biomechanical requirements. For the test bench design as shown in Figure 7a, the prosthesis was fixed on a testbench, a stiffness of 5000 N/m spring connection at distances of 20 cm from the center of the rotary joints, and the equivalent torsional stiffness is 200 Nm/rad. Angular displacement command described as seen (13) was conducted.

**Figure 8:**
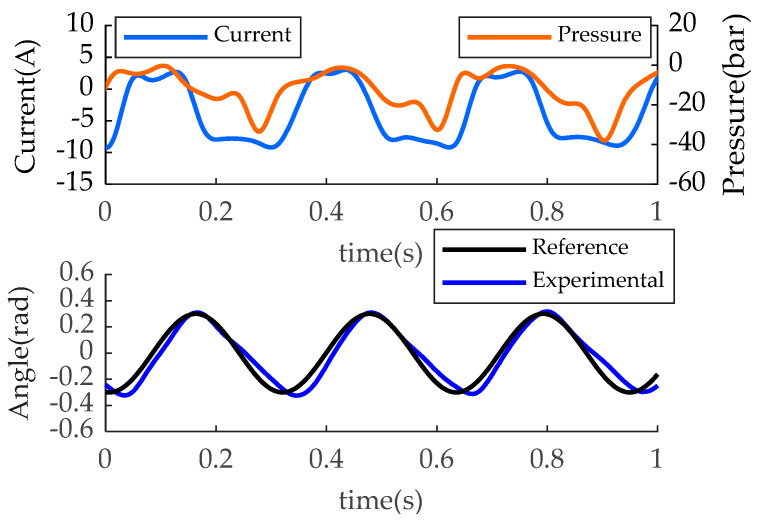
Sensor data of the loading test.

**Figure 9:**
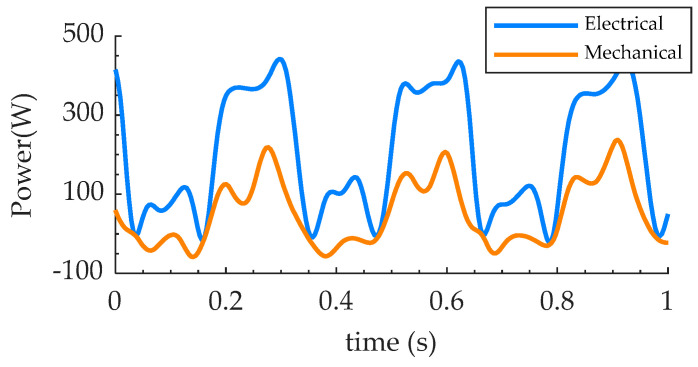
Mechanical power and electrical power of the loading test.

The experimental load trajectory is shown as the turquoise line in Figure 7b. The red point is the maximum power point on the trajectory (218 W, 52 Nm, and 4.2 rad/s), which is slightly larger than the target maximum power point of the orange line (178 W, 51 Nm, and 3.7 rad/s). The red translucent area is the area covered by the experimental maximum power point, within which the prosthesis can reach any operating point. Together with the red area, the experimental trajectory contains the orange curve inside. Therefore, from the perspective of load matching, the prosthesis can meet the requirements of the blue line corresponding to the human body. As shown in Figure 8, the angles of the joints can be tracked, but asymmetrical errors occur. This is because the controller adopts position control mode during the load test, and the spring force is applied to the prosthesis as a disturbance. From the experimental results, it does not affect the performance validation. The current ranges from −9.2 A to ~2.8 A, which is within the driver capacity limit (15 A). The fluctuation trend of current and pressure is basically the same, but the pressure sign stays negative and the current sign changes. This is mainly due to the inertial load of the motor and pump. With an average electrical peak power of 430 W and an average mechanical peak power of 200 W, the output power of the EHA system meets the requirements of the PEA, as shown in Figure 9. The electrical power shows two significant peaks (430 W and 120 W).

## 5. Discussion

According to the above experiments, the prosthesis can meet the load profile with a peak torque of 120 Nm, a maximum speed of 4 rad/s, and a fast recover time of 0.2 s, which is acceptable for a 75 kg male. We emphasize the importance of satisfying the load profile, i.e., the demands of the ankle joint at every moment in the gait cycle, rather than the performance of the prosthesis itself. Therefore, we analyzed the motor model and design the prosthesis parameters with biomechanical data as the target load. A situation can be seen in [13], where the maximum power of the ankle was 552 W, but it cannot provide enough torque compared with the biomechanical data. The load profiles were also not matched in comparable active EHA prostheses [21,22].

From the perspective of power density, the mass of the prosthetic prototype is 2.6 Kg. Although meeting the requirement, the mass can be reduced to a considerable extent on the valve block, with the help of metal 3D printing. The gear pump is possible to be integrated with the motor as one part, so the power density ratio of the prosthesis can be improved.

For EHA system, the parallel spring reduces the torque requirement and therefore the pre-charge pressure. During this experiment, the pressure difference between the two chambers at maximum load did not exceed 40 bar, so a pre-charge pressure of 20 bar was sufficient. It also greatly reduces the flow of external leaks and reduces the requirement for seals. For EHA systems without springs [21], a higher pre-charge pressure is required, which significantly increases the complexity of the sealing device.

From an overall design point of view, different choice of motors may lead to various designs. In Section 2.4, we obtained an optimization result of parallel spring in line with [15] which used a motor having similar characteristics to ours. In the selection of motor parameters, the choice of a motor with low inertia and high torque constant can help reduce motor current, thereby reducing motor energy consumption and improving efficiency. The inertial load was obvious, although we only applied an external elastic load, which came mainly from the inertial load of the motor. According to Figure 8, the first current peak was very close to the phase of the mechanical peak, while the second peak was close to the phase of the positive peak of the current, so it was mainly caused by inertial load. This illustrates the importance of choosing a small inertia motor, which is also mentioned in [15].

PEA can reduce motor torque requirements as shown in Figure 6, avoiding motors from overcurrent. The optimization result of [13] is a series spring, largely because they choose the torque motor as the power source where the SEA was designed to compensate the speed requirement. In terms of power, we generally consider that torque motor is suitable for SEA, while the high-speed motor is suitable for PEA. A single spring should be chosen as far as possible, rather than the combination of SEA and PEA, otherwise it increases the mass of the system and increases the difficulty of control. If the optimization result is always the combination of SEA and PEA, the main reason may be the unreasonable setting of transmission ratio.

## 6. Conclusions

For the EHA prosthesis with PEA as the actuation concept, we conducted a top-down design and optimized the three parameters of transmission ratio, spring stiffness, and equilibrium point after selecting the motor, and then proposed the mechanical structure design and control system design of the prosthesis. In order to verify the driving capability of the designed EHA prosthesis, we initially conducted rapid response experiments and loading experiments on the test bench, and the results showed that the modified prosthesis could meet the output capability requirements for level walking at normal speed. Conducting level walking experiments is the next step planned to verify the performance of the control system as well as PEA in walking. In addition, the prosthesis designed in this study is only suitable for level walking and a larger ROM and adjustable springs are required to accommodate more active tasks. Furthermore, we would like to emphasize that EHA can combine the properties of mechanical systems with hydraulic systems to achieve more forms of actuation concept. The literature [27] proves that a hydraulic damping prosthesis is more helpful to the COT of the human body than the ESR prosthesis. The literature [28] shows that adding series damping may be more helpful to the human body during the process of descending stairs. Therefore, this study initially verified the output performance of EHA. Combined with previous studies, the author believes that EHA provides more ideas in the design of wearable devices.

## Figures and Tables

**Table 1:** Performance metrics of the prosthesis.

ROM	Max Speed	Max Torque	SW Period
40° (−20°~20°)	4 rad/s	1.6 Nm/Kg	0.4 s

**Table 2:** Motor parameters of the Eci-40 Maxon brushless DC motor.

Parameters	Value
Torque constant $K_{t}$	0.091 Nm/A
Speed constant $K_{b}$	105 rpm/V
Terminal resistance R	1.01 Ω
Terminal inductance L	0.995 mH
Motor inertia $J_{m}$	44 gcm^2^
Friction coefficient $v_{m}$	5.21 × 10^6^ Nms/rad
Maximum permissive speed $n_{\max}$	8000 rpm
Maximum continuous current $I_{cont}$	2.39 A

**Table 3:** Parameters used in the simulation. *U*_max_ and *I*_max_ are the capacity of Escon 50/5. *J_p_* is the inertia of VIVOIL reversable pump. *η* was selected based on experience.

$U_{\max}$	$I_{\max}$	$J_{p}$	η
48 V	15 A	7 gcm^2^	70%

**Table 4:** Simulation result of the optimization.

$k_{p}$	$\theta_{eq}$	$P_{diver}$	$P_{PS}$	$P_{DD}$	$E_{diver}$	$E_{PS}$	$E_{DD}$
500 Nm/rad	−2°	348.6 W	208.3 W	524.7 W	18.3 J	29.1 J	64.2 J

**Table 5:** Main components in the powered ankle prosthesis.

Main Component	Features	
Maxon ECi-40 (P/N 488607) Brushless DC Motor	Nominal Voltage	48 V
	Rated Power	100 W
	Nominal Speed	5000 rpm
Escon 50/5 Servo Controller	Nominal Voltage	10–50 V
	Maximum Output Current	48 A
VIVOIL Reversible Gear Pump	Displacement	0.92 cc/rev
Integrated Ankle Joint Cylinder	Working Area Movement Range	6.5 cm^2^ 35°
Carbon fiber Sole	Poisson’s ratio	0.31
	Elastic modulus	2.1 × 10^5^ MPa

**Table 6:** Parameters of the elastic load and the loading track.

$k_{l}$	$\theta_{o}$	ω
200 Nm/rad	0.3 rad	20 rad/s

## Data Availability

The datasets generated during and/or analyzed during the current study are available from the corresponding author upon reasonable request.
